# Compliance and Adherence to Enteral Nutrition Treatment in Adults: A Systematic Review

**DOI:** 10.3390/nu11112627

**Published:** 2019-11-02

**Authors:** Alicia Gea Cabrera, María Sanz-Lorente, Javier Sanz-Valero, Elsa López-Pintor

**Affiliations:** 1 Department of Engineering, Area of Pharmacy and Pharmaceutical Technology, Universidad Miguel Hernández, 03550 Alicante, Spain; ali_ftca@hotmail.com; 2 Department of Public Health and History of Science, Universidad Miguel Hernández, 03550 Alicante, Spain; msanzlor@gmail.com (M.S.-L.); jsanz@umh.es (J.S.-V.); 3 Instituto de Salud Carlos III, Escuela Nacional de Medicina del Trabajo, 28029 Madrid, Spain

**Keywords:** treatment adherence and compliance, enteral nutrition, attitude to health, nutritional support, adult

## Abstract

Objective: To review the scientific literature that has verified and/or assessed compliance and adherence to enteral nutrition (EN) in adult patients. Method: This study involved a critical analysis of articles retrieved from MEDLINE (PubMed), The Cochrane Library, Embase, Scopus and Web of Science using the terms “Treatment Adherence and Compliance” and “Enteral Nutrition”, applying the filters “Comparative Study” or “Clinical Trial”, “Humans” and “Adults”. Date of the search: 25 October 2018. Results: A total of 512 references were retrieved, of which 23 documents were selected after applying the inclusion and exclusion criteria. The techniques measuring adherence to EN were determined by dietary intake, self-reporting, counts of leftover containers or presence of complications; however, in no case were validated questionnaires used. The time and periodicity of the assessment presented very heterogeneous results, with measurement predominantly being done at the beginning and at the end of the study. The best adherence rates were obtained in hospitalized patients (approximately 80%). Conclusions: Frequent and regular monitoring of the adherence of patients under prolonged treatment with EN is necessary, and the use of measurement techniques that allow obtaining information on the causes of non-adherence facilitates early interventions to optimize treatment outcomes. Patient and/or caregiver education in the management of EN and the intervention of the community pharmacy in monitoring patients can be key to improving the adherence to EN.

## 1. Introduction

Enteral nutrition (EN) is a treatment consisting of administering, through the digestive tract, nutrients necessary to maintain an adequate nutritional status in patients who cannot meet their nutritional needs orally due to their clinical situation but whose digestive tract still functions for digestion and absorption [1]. However, the efficacy of this nutrition is determined by several factors: some are intrinsic to the treatment itself, others are derived from the disease and other are determined by adherence behavior [2].

Concern about adherence gained importance in the second half of the 20th century, when advances in the health sciences made treatments safer and more effective; however, lack of compliance continued to lead to unresolved indications and the appearance of concomitant problems [2]. This complex situation continues to be a current problem, probably because in recent years, there has been a progressive increase in the incidence of chronic diseases that lead to the the coexistence of simultaneous treatments in the same patient for a prolonged period [3].

The concept and definition of adherence have been widely discussed, which is why the World Health Organization (WHO) [4] issued, in 2003, a report on “Adherence to long-term therapies”, which showed that the definition considered until then was strictly focused on adherence to pharmacological treatment or medical indications and did not include any other type of intervention or recommendation regarding changes in lifestyle (hygiene, exercise, nutrition, etc.), leaving out a large number of patient behaviors that compromised the evolution of their pathology and their well-being. Therefore, the WHO proposed in the Adherence Project [5] to update the concept as “The extent to which a person’s behavior—taking medication, following a diet, and/or executing lifestyle changes, corresponds with agreed recommendations from a health care provider”.

According to the Medical Subject Headings (MeSH) of the US National Library of Medicine (NLM), the definition for “Treatment Adherence and Compliance” would be: the extent to which the patient follows prescribed treatment such as keeping appointments and schedules and medication adherence for desired therapeutic outcome. It implies active responsibility shared by patient and health care providers (NLM MeSH Homepage: https://www.ncbi.nlm.nih.gov/mesh).

The American and European societies of enteral and parenteral nutrition showed, in their clinical guidelines, the existence of discrepancies between the amounts prescribed and those received by patients who were administered artificial nutrition with feeding tubes [6,7]. Van den Broek et al. [8], in a study in a hospital setting, assessed adherence during the admission period. Although these results can be considered an approximation for assessing adherence, it cannot be assumed that there is a real, or even approximate, vision of what happens with patients who have had EN prescribed for a long time, especially in the home environment, where the controls, registries, pumps, dedication and interest in complying with the established nutritional regimen is, undoubtedly, highly variable [2].

Consequently, the objective of this study was to review the scientific literature that has verified and/or assessed compliance and adherence to EN in adult patients.

## 2. Materials and Methods

### 2.1. Design

Cross-sectional descriptive study and critical analysis of the works systematically retrieved.

### 2.2. Source of Data Collection

The data were obtained from direct consultation and access, via the Internet, to the following bibliographic databases in the field of health sciences: MEDLINE (via PubMed), The Cochrane Library: The Cochrane Central Register of Controlled Trials (CENTRAL) and The Cochrane Database of Systematic Reviews (CDSR), Embase, Scopus and Web of Science.

### 2.3. Information Search

To define the search terms, the thesaurus developed by the US National Library of Medicine was referred to.

The search strategy was planned around three domains:

- Population: adults with an age equal to or greater than 19 years;
- Intervention: EN;
- Outcome: known compliance and adherence to treatment and the method used for its assessment.

For this, the search syntax was generated using the Boolean intersection of two equations: (Equation (1)) and (Equation (2)).

Equation (1): Treatment Adherence and Compliance.

“Treatment Adherence and Compliance” [Mesh] OR “Treatment Adherence and Compliance” [Title/Abstract] OR “Patient Acceptance of Health Care” [Title/Abstract] OR “Patient Compliance” [Title/Abstract] OR “Patient Dropouts” [Title/Abstract] OR “Patient Participation” [Title/Abstract] OR “Patient Satisfaction” [Title/Abstract] OR “Patient Preference” [Title/Abstract] OR “Treatment Refusal” [Title/Abstract] OR “Patient Acceptance of Health Care” [Title/Abstract] OR “Patient Dropouts” [Title/Abstract] OR “Patient Adherence” [Title/Abstract] OR “Patient Cooperation” [Title/Abstract] OR “Patient Non-Compliance” [Title/Abstract] OR “Patient Non Compliance” [Title/Abstract] OR “Patient Nonadherence” [Title/Abstract] OR “Patient Noncompliance” [Title/Abstract] OR “Patient Non-Adherence” [Title/Abstract] OR “Patient Non Adherence” [Title/Abstract] OR “Treatment Compliance” [Title/Abstract] OR “Treatment Compliances” [Title/Abstract] OR “Therapeutic Compliance” [Title/Abstract] OR “Therapeutic Compliances” [Title/Abstract]

Equation (2): Enteral Nutrition.

“Enteral Nutrition” [Mesh] OR “Enteral Nutrition” [Title/Abstract] OR “Enteral Feeding” [Title/Abstract] OR “Force Feeding” [Title/Abstract] OR “Force Feedings” [Title/Abstract] OR “Tube Feeding” [Title/Abstract] OR “Gastric Feeding Tubes” [Title/Abstract] OR “Gastric Feeding Tube” [Title/Abstract].

The following filters were applied: “Humans”, “Adult 19+ years” and “Comparative Study” or “Clinical Trial”.

The final search equation was developed for use in the MEDLINE database, via PubMed. Subsequently, this strategy was adapted to the characteristics of each of the other databases consulted and was completed by examining the bibliographic references of the selected articles.

The search was performed from the first available date until the day of the last query of the databases (initial search in MEDLINE 25 October 2018).

Additionally, a search using a complementary strategy was conducted to reduce the possibility of publication bias by searching the reference lists of relevant guidelines. Furthermore, experts in the domain were contacted by mail to avoid issues regarding possible grey literature (materials and research produced by organizations outside of the traditional commercial or academic publishing and distribution channels).

### 2.4. Final Selection of Articles

The records that met the following inclusion criteria were accepted for review: clinical trials or comparative studies that fit the objectives of the search and were published in peer-reviewed journals.

A selection of references first based on title/abstract and after on full-text review was performed. Articles were screened based on the availability of the complete text, the existence of a causal relationship between treatment adherence and EN, and the inclusion of adults in the intervention (EN). Any article that did not meet these criteria was excluded. 

The selection of relevant papers was performed independently by two authors: A.G.C. and E.L.P. To include the studies, it was established that the valuation of the concordance between these authors (kappa index) must be greater than 60% [9]. Provided this condition is fulfilled, possible discrepancies were solved through consultation with the author J.S.V. and subsequent consensus amongst all the authors.

### 2.5. Quality of Reporting of the Selected Documents 

To assess the quality of reporting of the selected documents, the CONSORT (Consolidated Standards of Reporting Trials) statements were used [10]; the checklist contains a list of 25 essential aspects that should be described in the studies. One point was assigned for each item present (if not applicable, it was not scored). When an item was composed of several points, the points were assessed independently, giving the same value to each point and then averaging them (the final result of that item), so that in no case was it possible to score more than 1 point per item.

### 2.6. Obsolescence

To inform of the actuality/obsolescence of the clinical trials selected for the review, the Burton-Kebler half-life (median age) and Price Index (percentage of articles less than 5 years old) were calculated.

### 2.7. Data Extraction

Continuous control for data correction was ensured by using double-entry charts that allowed deviations to be detected and corrected by making a new query of the originals. Data extraction was carried out independently by A.G.C. and E.L.P., and M.S.L. was responsible for the verification of the tables.

### 2.8. Study Variables

- The studies were grouped according to the variables studied to systematize and facilitate the interpretation of the results, considering the following data:
- Author: the first author of the article was selected;
- Year: year of publication of the article;
- Design: procedures, methods and techniques through which the article was accepted for review. In this case, only clinical trials or comparative studies were accepted;
- Population studied: Adults undergoing EN intervention;
- Country: location where the intervention took place;
- Pathology: disease of the population for which the intervention was performed;
- Type of nutrition: if total/exclusive enteral nutrition (EEN), when the only source of food is formula-based, or partial (PEN);
- Form and frequency of administration: route through which EN was administered and its periodicity;
- Technique for measuring adherence: procedure used to determine adherence to EN;
- Outcome observed: causal relationship derived from the intervention (administration of EN).

## 3. Results

With the described search criteria, 512 references were retrieved: 53 in MEDLINE, 71 in the Cochrane Library, 47 in Embase, 43 in the Web of Science and 298 in Scopus.

**Figure 1:**
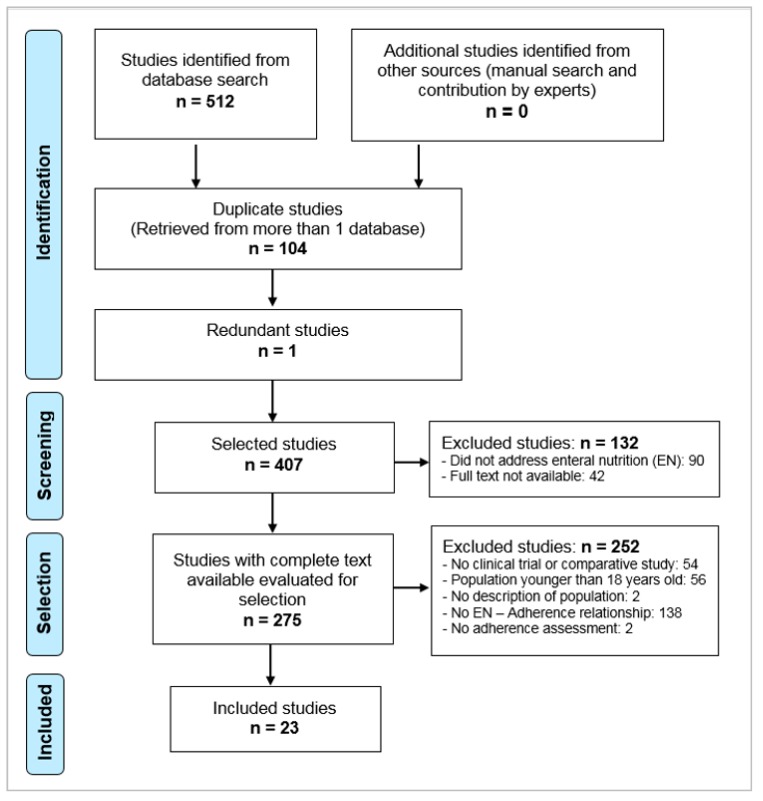
Selection procedure of the studies.

After eliminating duplicates, applying the inclusion and exclusion criteria, consulting the bibliographies of the selected articles and consulting with experts (Figure 1), 23 documents were selected [11,12,13,14,15,16,17,18,19,20,21,22,23,24,25,26,27,28,29,30,31,32,33] (Table 1). Two articles by Brown et al. [15,34] were selected; although they presented different objectives of study, the population analyzed was the same, and adherence results were not different between them, and therefore, only one was accepted for review [15].

There were two studies that were initially included in the review [35,36], but after careful review of their content, patient compliance with EN had not been assessed. Goh et al. [35] assessed adherence to the recommended initiation of EN in Parkinson’s patients at risk of dysphagia, without providing compliance data. Peerawong et al. [36] studied adherence to radiation therapy and chemotherapy in patients with prophylactic gastrostomy tubes; however, they did not assess adherence to EN treatment during the 52-day monitoring period. Because the two aforementioned investigations were retrospective studies, information on adherence was probably available. This lack of knowledge on adherence could lead to an underestimation of treatment effects.

When evaluating the quality of the selected articles using the CONSORT questionnaire, the scores varied between 7 and 21 (compliance with 25 of 28 items, or 84%), with a median of 11.5 (see Table 2).

The agreement between authors in the selection of articles, measured by the Kappa coefficient, was 70.85% (*p* < 0.001). Twenty-three articles presented obsolescence, according to the Burton-Kebler Index, being 6 years old, with a Price Index of 47.83%.

Study design included seven comparative studies [11,13,22,23,25,30,31] and 16 clinical trials [12,14,15,16,17,18,19,20,21,24,26,27,28,29,32,33], all written in English and developed in 10 different countries, with the United Kingdom [17,19,20,21,29,32,33] and Japan [11,23,26,28,30,31] contributing the most work.

The articles studied a highly variable number of participants, from *n* = 1197 [17] to *n* = 22 [22], focused predominantly on males according to the male/female ratio. The average age was approximately 60 years in most studies, except for the studies by Hirai et al. [11] and Wall et al. [12] with patients with Chron’s disease, who were approximately 20 years old. The most common underlying pathology was neoplasia, nine studies [15,16,20,21,23,24,26,29,30], and most of the research was carried out in a nonhospital setting (non-hospitalized patients, PNH), 16 studies [11,12,13,15,16,19,21,22,24,25,26,27,28,29,30,31].

The longest recruitment period was that of the study by Healy et al. [16], from January 2011 to December 2014. The longest monitoring time was that of the study by Hirai et al. [11], which was 2 years.

Regarding EN type, 11 studies involved partial enteral nutrition (PEN) [11,13,15,16,19,21,23,25,28,29,32], five studies involved exclusive enteral nutrition (EEN) [14,18,24,27,33] and seven studies involved different combinations of PEN and EEN [12,17,20,22,26,30,31].

In 10 studies, the main route of administration was through a tube (gastrostomy, nasogastric, jejunostomy, etc.) [13,14,15,17,18,21,22,24,27,33]; in seven studies, the route was either oral or through a feeding tube [11,16,20,23,26,28,30]; and in six articles, the route was exclusively oral [12,19,25,29,31,32]. However, the choice of administration form depended on the underlying pathology [11,12,21,22,24,27,28,29,30,31,33,35], the treatment (e.g., chemotherapy) [15,16,26,36], prior surgery [13,15,20,23,32] and individualized patient requirements [14,17,18,19,25].

### 3.1. Adherence Measurement Methods

Adherence was assessed by measuring intake in 15 of the 23 articles included. Intake was determined by dietary intake, self-reported intake of nutrients or energy by the patients themselves [12,13,15,16,20,21,25,29] or directly by the project staff [17,19,23,32]. Two studies [26,30] measured the consumption of kcal and protein without indicating the form of administration, and Hirai et al. [11] related adherence with continuing with the prescribed amount (versus amount ingested) during the study period.

In eight studies [14,15,16,19,20,29,32,33], adherence was assessed based on the number of containers returned by the patient (or self-reported intake) and the number of prescribed containers: (containers returned or reported as ingested/prescribed containers) * 100.

In 14 studies [12,13,16,17,19,20,21,22,23,24,25,26,30,33], markers of nutritional status and/or anthropometric parameters were assessed as measures of the outcome of the interventions. Other studies [18,22,24,27,28] assessed the presence of mechanical complications of EN and/or gastrointestinal side-effects of EN adherence, such as nausea, vomiting or diarrhea, an aspect also recorded in studies [11,12,14,15,16,21]. Finally, in four of the included studies, patients received education about EN (dietary advice and/or feeding tube management and nutritional supplements) at the beginning of the investigation [21,22,28,31].

### 3.2. Periodicity and Time of Measuring Adherence

Regarding the timing and periodicity with which adherence was assessed, the results are very heterogeneous. Benton et al. [13] and Sukkar et al. [22] performed two single measurements, one at the beginning and one at the end of the study. In the rest of the studies, there was very variable monitoring, from daily to weekly or with a periodicity of up to 6 months.

In nine studies, daily measurements of compliance with EN by the patient were performed; six of the studies were performed with hospitalized patients with a monitoring period of less than 1 month [14,17,18,20,23,33]. The studies by Miyata et al. [26] and McCough et al. [29] were performed with non-hospitalized patients, with adherence monitored for 17 days and 5 weeks, respectively. In the study by Kraft et al. [25], monitoring of adherence was carried out daily for 6 months through telematic means.

In general, studies with monitoring periods of 3 or more months [11,16,19,21,24,28] recorded measurements every 2 [11] or 3 months [19,28], or recorded two measures close together: one at the beginning and one in the first weeks, and the next at 6 months [21,24]. Brown et al. [15], performed [12,16,30,32] weekly measurements for 3 months.

Studies less than 3 months long [12,16,30,32] recorded measurements weekly [32], every 2 weeks [12,30] or at 1 month [16].

### 3.3. Adherence Rates and/or Compliance with EN Protocols

In 11 of the studies reviewed [11,12,13,14,15,16,17,19,23,30,33], quantitative data were provided on the rate of adherence to EN or the degree of compliance with the established EN protocol.

In general, studies measuring the adherence of hospitalized patients to EN [14,16,17,23,30,33] presented rates above 80%, with the exception of Lawson et al. [32], who obtained a median compliance of 14.9% in patients undergoing orthopedic surgery who were followed for 14 days, noting that none of the patients were 100% adherent, taking half of the prescribed volume in half of the indicated time, and that during monitoring, 76% of the patients made some readjustment in the type of supplement to adapt it to their preferences.

In the studies performed with non-hospitalized patients, the adherence rates were lower. In the studies by Hirai et al. [11], Wall et al. [12], Benton et al. [13] and McCough et al. [29], adherence did not reach 50%. Brown et al. [15], in their clinical trial conducted in patients with head and neck cancer, found an adherence rate of 51% in patients who were introduced to EN in the early (prophylactic) phase, highlighting the progressive increase in adherence in the first 4 weeks, which they justified as a period of adaptation to the feeding tube. Stow et al. [19], in a controlled clinical trial conducted with patients at risk of severe malnutrition living in adult care homes, found 74% adherence at 3 months and 67% at 6 months, where 86% of patients met at least 50% of the requirements. 

The work by Hamza et al. [20] and Bowrey [21] assessed EN ingestion but did not provide data on adherence. Takagi et al. [28] indicated that adherence was similar between the compared groups but did not specify how and when it was assessed. Sukkar et al. [22] only recorded complications in the maintenance of the feeding tube, without monitoring compliance with EN; however, they found that 9% of patients abandoned treatment due to low compliance.

Healy et al. [16], Zhao [18], Sadasivan et al. [24] and Tsukikawa et al. [31] assessed patient satisfaction and/or quality of life, both variables related to adherence.

## 4. Discussion

The present review demonstrated, in one way or another, the special interest in knowing compliance and adherence to nutritional treatment.

Considering that both clinical trials and comparative studies have been included in this review, it is likely that the CONSORT questionnaire was not the most suitable for evaluating the quality of articles with comparative designs, which is why the value obtained in the results was not as high as expected for rigorous studies. Although systematic reviews should be based on studies with monitoring protocols and designs that guarantee the greatest scientific rigor, in the present analysis, all articles investigating the analyzed topic were included to achieve maximum representation in the results. Restricting the review to clinical trials and comparative studies was decided so as to search for a consistent cause-effect relationship [37].

The low obsolescence of the studies included in the review indicated the validity and timeliness as well as the interest in the chosen topic; the data obtained (Price Index and Burton Kebler index) indicate lower obsolescence than the usual bibliometric results in the field of nutrition sciences, demonstrating that this is a newly emerging area [38].

The language of the included studies was as expected, with all papers analyzed written in English. This language is preferred for the publication of the majority of articles because publication in a different language decreases visibility, the impact factor and citations. In addition, the number of anglophone journals contained in the databases is currently very high [39].

The population included in the studies was notably older, mainly because the pathology with the greatest presence was neoplasia, mainly treated in the home setting, where studies related to this circumstance already exist [1].

The recruitment and monitoring periods were considered adequate and consistent with what was observed in previous systematic reviews [37], a requirement that the selected studies met except for the articles by Deane et al. [14], Harvey et al. [17], Zhao [18] and Shirakawa et al. [23], whose study durations were too short to adequately assess pharmacotherapy-therapeutic monitoring. However, as has been investigated, nutrition monitoring of patients was directly related to improvements in nutritional status [40].

The clear predominance of PEN over EEN followed the recommendations of the different clinical guidelines, which clearly state that before establishing enteral treatment, the possibility of consuming normal foods (natural, prepared or processed) should be assessed. If this is not possible, enteral formula-based foods should be used. Only in clinical situations where the oral route was compromised was EEN chosen [41,42]. These same recommendations served as the basis of choosing the route of EN administration; however, the form of administration depended, as seen in the results, on the underlying pathology and its treatment (e.g., chemotherapy) and on individual patient requirements.

### 4.1. Adherence Measurement Methods

In the majority of the studies reviewed, the assessment of adherence to EN was performed by indirect techniques, such as dietary intake (self-report of intake by the patient) or the number of leftover containers and its relation with the prescribed number provided. Both techniques offer an approximation of an individual’s behavior regarding medication. However, according to Lehmann et al. [43], these procedures have limitations. In principle, the questionnaires used to measure intake should be validated. Wall et al. [12] used electronic self-reporting of intake by the patient to evaluate adherence, recognizing as a bias that the questionnaire was not validated in adults. In the rest of the studies, no details on the validation of the tool used were provided. Furthermore, the amount that the patient claims to ingest may be influenced by forgetfulness or be distorted, especially if recorded by someone other than the patient, for example, the caregiver [44], or by a lack of trust in the relationship between the patient and the healthcare professional. The count of leftover containers, meanwhile, is a static measure that does not reflect the daily variability of adherence. There are also risks of false positives due to patients emptying the containers before returning them, which is common if the patient feels watched [43].

Lack of adherence can be due to multiple causes, such as the presence of barriers or beliefs of the patient, ignorance and forgetfulness or carelessness [45]. The multifactorial nature of the problem means that there is no ideal method for measuring adherence. Therefore, to increase the validity and reliability of the adherence data collected, combining more than one technique is recommended depending on the information desired from the patient. In this sense, the questionnaires, despite their limitations, constitute the simplest method and are used both in research and epidemiological studies and in clinical practice due to their simplicity, ease of application and ability to guide toward the possible cause of lack of adherence [43]. A relevant fact detected in this review is that none of the studies evaluated used specific questionnaires to measure adherence to EN, such as that proposed by Wanden-Berghe et al. [2]. If in more than half of chronic patients this type of nutrition is prescribed [46], it seems reasonable to have a validated instrument that allows for managing nutritional adherence.

In the majority of the studies, the anthropometric and biochemical parameters and nutritional state of the patient were assessed. These results are usually part of the routine monitoring of patients treated with EN [41,42]; however, their use as surrogate markers of adherence is limited because they can be affected by other factors, not only by the behavior of the patient but also by the adequacy of medication and biological, genetic or environmental factors [43].

Additionally, some studies used the presence of mechanical complications of EN and/or gastrointestinal side-effects (nausea, vomiting or diarrhea) as indicators of adherence. These problems are also frequently monitored in patients with EN [47] and are related to the tolerability of the formula, and while their appearance may cause treatment withdrawal, they may not be the only cause of non-adherence. In addition, good tolerance does not guarantee patient adherence, and therefore, should not exempt the need to monitor adherence. Although several of the studies reported complications and adverse effects, it is noteworthy that for only four patients were the patient and/or caregivers previously educated and trained in the management of EN. This fact is relevant; EN may entail “complex” drugs, which are those whose use requires special skills by the patient and may be a potential source of error, such as inhalers [48]. In this type of treatment, as in EN, adherence is influenced by multiple factors (complexity of the delivery devices, administration guidelines, patient beliefs or sociocultural factors), and patients or their caregivers should be educated to maximally reduce complications and facilitate their independence and self-sufficiency as much as possible [41]. In this sense, patient empowerment is a key element to improving adherence [49].

### 4.2. Periodicity and Time of Measuring Adherence

Compliance monitoring was carried out with a highly variable periodicity, from studies where monitoring was performed daily to others that conducted weekly monitoring or with a periodicity of up to 6 months.

If the studies lasting less than 3 months are eliminated and those conducted in inpatients where controls were more frequent and, often, the professionals administered EN, the remainder of studies assessed adherence with less frequency: every 2–3 or even 6 months, with no interventions to improve adherence. This would explain the low adherence rates found in some studies. This periodicity coincides with the minimum frequency of 3 months with which it is recommended that EN be assessed [41,42].

However, therapeutic adherence has a dynamic behavior, probably less stable than nutritional status or biochemical parameters, which means that it must be reevaluated with a certain frequency to prevent possible variations over time. Gearing et al. [50] proposed up to six phases of adherence to treatment: initiation, trial, partial acceptance, intermittent treatment adoption, premature discontinuation and total adherence.

Kraft et al. [25] carried out a comparative study in patients at risk of severe malnutrition, where monitoring was performed telematically daily for 6 months. The authors did not present adherence figures; however, this system made it possible to receive alerts such as that the patient “has not taken the oral nutritional supplement”, “did not like the taste of the oral nutritional supplements” or “took only a little bit”. In this sense, “telehealth” and the use of telematic means is a way of maintaining regular contact with health professionals and facilitating the monitoring of adherence to prescribed treatments by collecting vital signs and data on intake and sharing information among patients, caregivers and professionals involved so that everyone has access to information in real time [51], thus allowing the implementation of early interventions. In any case, the limitations of this form of EN monitoring should be assessed because the authors reported difficulties in recruiting patients in the study because they did not understand the importance of monitoring EN or were not familiar with electronic devices.

In any case, there is no ideal frequency of measurement, but the ideal time to assess adherence would be the moment when this type of treatment is dispensed in chronic non-hospitalized patients. Evaluating how patients’ pattern of use departs from the methodology of dispensation would detect situations of non-adherence and allow for interventions to improve adherence [52]. In this sense, the community pharmacist is in the ideal position to carry out this task [53]. A recent systematic review and meta-analysis of 771 articles on adherence interventions published in 2017 suggested that the most effective interventions are those performed face-to-face with the patient and by pharmacists [54]. There are other studies that support the contribution of community pharmacists to improving adherence [55]. Therefore, the community pharmacist could take on the role of being responsible for adherence monitoring in each dispensation, that is, approximately once a month.

### 4.3. Adherence Rates and/or Compliance with EN Protocols

In relation to the adherence rates found, the results of this review showed a clear differentiation between studies conducted in a hospital setting and those conducted with non-admitted patients, with influence in both cases of patient monitoring time. The best adherence rates, above 80%, were obtained in hospitalized patients and in short monitoring studies, such as those by Deane et al. [14], Healy et al. [16], Harvey et al. [17], Shirakawa et al. [23] and Park et al. [33]. This is probably because patients are better managed by professionals in a hospital environment where monitoring is simpler and is usually part of routine practice, something that also occurs when the patient is in an adult care home, as demonstrated by Stow et al. [19], who obtained adherence rates greater than 70%.

Lawson et al. [32], on the contrary, constitute the exception, finding an adherence rate lower than 15%. The explanation for this low adherence in the hospital setting could be due to the method of intensive monitoring of adherence, probably the most exhaustive of all the studies reviewed: the patient had to sign when given the supplement or if it had been rejected and the reason; the patients, nurses and staff recorded the amount ingested for each supplement; dietitians completed a weekly review; discussions regarding progress were held with the patient; total supplements ingested and/or rejected were recorded; and patients self-reported the food and beverages consumed outside of hospital catering. Through this methodology, the authors were able to accurately evaluate the adherence of each patient, calculate the volume consumed for each container and the time during which the instructions were followed, and determine the patient’s willingness to make changes regarding the type of supplement and record discontinued treatment mutually decided upon by the patient and professional. These results showed that in EN monitoring, the patient’s preferences should be taken into account, as the benefit of nutritional supplementation depends on the acceptance and compliance of patients.

In contrast, when the patients were at home, the situation changed. The pattern of adherence observed in non-hospitalized patients with a monitoring period of 3 months or more was similar to that of patients with chronic pharmacological treatments, where adherence was less than 50% [4], altering the outcome of the interventions. Thus, Hirai et al. [11] found no significant differences in the remission rate of Crohn’s disease between control and intervention groups, attributing this result to the lack of adherence to EN by the intervention group, where only 11/37 patients met the prescribed caloric requirements. Brown et al. [15] obtained a 51% adherence rate, which was lower than expected, in patients in whom early intubation was initiated. However, patients in this group were more adherent than those in the control group once treatment started (58% versus 38%), resulting in less significant weight loss in patients from the intervention group, which the authors attributed to greater adherence.

Furthermore, Wall et al. [12] found an EN protocol abandonment rate close to 40%, and Benton et al. [13] found that less than 50% of patients with jejunostomy met predefined caloric and protein requirements at 42 days of treatment. McGough et al. [29], in patients with gynecological cancer, found a progressive decrease in adherence over time, decreasing from 92% in week 1 to 46% in week 5, a downward progression that was also reflected in the studies by Stow et al. [19]. These data agree with what has been described for chronic pharmacological treatments, where it was estimated that during the first year, 1 in 2 patients discontinues treatment [56] or there is a progressive loss of adherence until the patient accepts the treatment and becomes familiar with it [57]. In the case of EN, in addition, the use of a feeding tube requires a period of adaptation, as suggested by Brown et al. [34].

The high adherence found by Piquet et al. [30] (close to 80%), was attributed to the fact that nutritional support was proposed very early by medical staff as an additional component of treatment. This is another important point. The lack of consideration of EN by patients and/or caregivers as part of the success of treatment may lead to relativizing its usefulness and thus compliance, a fact also discussed by Kraft et al. [25]. The professionals involved should educate patients that EN is not simply a food but that its consumption can help achieve the expected positive treatment outcomes.

### 4.4. Limitations of the Review

The Scopus database initially retrieved many works that were ultimately irrelevant, which could be due to the lack of indexing (the search was done in text format querying the title, abstract and keywords) and the impossibility of limiting the search by the type of article (restricted to clinical trials or comparative studies). This high document “noise” was previously observed in other systematic reviews [58,59]. Another important limitation of the present review was not being able to retrieve the full text of some articles because they were not digitized on the journal’s website or did not appear in the main journal collections and even could not be retrieved through the network of university libraries. Finally, with regard to the adherence results, it would have been interesting to summarize them by a quantitative value or to give a measure of central tendency; however, the few papers showing data regarding the percentage of adherence and the great heterogeneity in the different adherence measurement techniques hindered this calculation.

### 4.5. Critical Review by the Authors

Knowing the degree of adherence of patients is essential to evaluate the effectiveness and safety of treatment with EN. Currently, there is not an established adherence rate in EN that guarantees a therapeutic outcome, which means that patient achieve their nutritional needs; however, in accordance with Apolo et al. [60], we would consider an acceptable adherence rate the compliance of at least 70% of individual requirements. EN, due to its characteristics and complexity of management, could be compared to chronic treatment, which requires special monitoring and patient education in its management and where adherence should be reevaluated with a certain frequency to prevent possible variations over time, especially in patients not hospitalized for prolonged treatment. Increasing the periodicity of monitoring in combination with intake assessments and using specific questionnaires to detect causes of non-adherence are two recommended measures to detect non-adherence and to design interventions to improve the EN pattern of use. The community pharmacist, due to their closeness to the patients and being the person in charge of the periodic dispensing of EN formulas, could play an important role in improving the adherence of the patients to prolonged EN treatment. 

## 5. Conclusions

Given the above, it was possible to conclude that more frequent and regular monitoring of patient adherence to prolonged EN treatment is necessary as well as using measurement techniques to obtain information on the causes of non-adherence to facilitate early intervention to optimize treatment outcomes. Patient and/or caregiver education in the management of EN and the intervention of the community pharmacist in monitoring patients can be key to improving the pattern of use of EN.

## Figures and Tables

**Table 1:** Summary of the studies reviewed on compliance and adherence to enteral nutrition in adults.

Author, Year	Design	Population Studied	Pathology	Scope	Country	Period of Study	Type of Nutrition	Form and Frequency of Administration	Technique for “Measuring” Adherence	Observed Outcome
Hirai et al. 2019 [11]	Comparative: Prospective multicenter cohorts	*N* = 72 M/F: 52/20 Age: IG (18–59; median 27); CG (18–70; median 28 years)	Crohn’s disease in maintenance treatment with anti-TNF antibodies	PNH	Japan	Recruitment: July 2011–March 2014. Monitoring period: 2 years.	PEN	Oral or tube. Formula Standard ≥900 kcal/day	Doctor-patient interviews every 8 weeks. Measurement of compliance with the prescribed amount	Low adherence rate in IG. Only 11/37 patients were adherent to EN.
Wall et al. 2018 [12]	Prospective nonrandomized trial	*N* = 38 Age: EEN Group (15.8–38.4; median 23.3); PEN Group: (16.5–38.2; median 19.2)	Chron’s disease (active)	PNH	New Zealand	Recruitment: May 2013–December 2015. Monitoring period: 8 weeks.	IG: EEN + EEN CG: EEN + PEN	Oral. 2 weeks. EEN + 6 weeks EEN or PEN.	(1) Monitoring by dietitian at weeks 0, 2, 4, 6 and 8. (2) Record of nutritional and anthropometric markers of nutritional status (3) Assessment of ingestion from the electronic self-report of intake by patient (dietary intake).	Significant improvement of symptoms, nutritional and inflammatory markers at 2 weeks. No effect at 8 weeks
Benton et al. 2018 [13]	Comparative study of historical cohort	*N*: 30 Age: IG (48–77, median = 62); CG (50–78, median = 61)	Esophagectomy	PNH	Australia	IG: Recruitment: October 2014–November 2016; CG: historical cohort of patients undergoing surgery between January 2011 and December 2012. Monitoring period: 42 days	PEN	Form: JS. (1) IG: surgery, days 1–3: clear fluids; days 4-12 (discharge): soft diet + EN. Days 12–42: oral diet + oral EN (2) CG: surgery, days 1–3 EN; days 4–7 clear fluids + EN; days 8–12 (discharge): soft diet + EN on demand. Days 12–42 total cessation EN.	Measures at the beginning and at the end of the study; (1) Nutritional status; (2) IG: Telephone interview with dietician 1 week after discharge; (3) Dietary intake (3 days of patient reporting)	The calorie and protein requirements were not met at day 42 postsurgery in any group.
Deane et al. 2018 [14]	Randomized clinical trial	*N*: 84 M/F: 48/32 Age: CG (mean = 55 (SD 16)); IG (mean 60 (SD 17))	Critical patients	PH	USA	Monitoring period: 7 days	EEN	Form: Gastric tube. Frequency: Individualized determination of the requirements in each patient.	Adequacy of delivered daily amount (delivered/prescribed × 100)	No differences between groups in the increase in calories and proteins received by the critical patient.
Brown et al. 2017 [15]	Randomized clinical trial	*N*: 125 M/F: 112/13 Age: median 60 years	Neoplasia of the head and neck	PNH	Australia	Recruitment: September 2012 to June 2015. Monitoring period: NC	PEN	Form: GS. Frequency: IG: (Prophylactic phase: early intubation prior to surgery): EN+ oral intake. Clinical nutrition phase: IG: increment EN; CG: EN according to nutritional requirements	(1) Daily self-support of EN intake; (2) record of symptoms from nutritional impact; (3) Reasons for noncompliance. Weekly collection by the dietitian	Overall adherence to tube feeding was significantly greater in IG (58% versus 38% CG). Early tube feeding may improve patient adherence to clinically indicated feeding.
Healy et al. 2017 [16]	Randomized clinical trial	*N* = 191 (IG = 97; CG = 94). M/F = NC Age: Not available	Esophagus neoplasia	PNH	Ireland	Recruitment period: January 2011 to December 2014. Monitoring period: 6 months	PEN	G1: omega 3 EN, oral or NG G2 = standard EN. Frequency: G1 and G2 = 600 kcal/day 3 days before and 7 days after two 17-day cycles of chemotherapy + oral intake.	(1) Self-reporting of compliance by the patient and analysis of product dispensed and returned (recount). (2) Assessment of quality of life, (oncology generic questionnaire)	Adherence in patients at admission: 98%; at home: 96%. Mean days of EN lost: 1.1 (range 0 to 13 days). Differences in quality of life between the two groups.
Harvey et al. 2016 [17]	Randomized clinical trial	*N*: 1197 (EN group) M/F: 725/472 Age: 62.9 ± 15.4	Critical patients	PH	United Kingdom	Recruitment: June 2011–October 2013. Monitoring period: 5 days	NP vs EEN	Form: NG/ND EN. Frequency: 1365–2540 kcal/day.	Adherence to nutritional protocol, with sporadic monitoring visits. during the first 120 h.	Adherence rate = 97%. Patients in the EEN group were more likely to have complete days without nutritional support.
Zhao 2015 [18]	Randomized clinical trial	*N*: 126 M/F: IG = 39/24; CG = 37/26 Age: Mean = 52.8 ± 9.5 years	Patients admitted to the Intensive Care Unit	PH	China	Recruitment: January–December 2014. Monitoring period: 7 days.	EEN	Form: NG tube. Frequency: CG = 430 g vegan formula IG = 500 g improved EN formula: 50 mL/4 h	Questionnaire of patient satisfaction at hospital discharge.	Satisfaction greater in the IG.
Stow et al. 2015 [19]	Randomized clinical trial	*N*: 93 M/F: 82% female. Age: 65 years on average	Assisted living home care patients at risk of protein-energy malnutrition (MEP)	PNH	United Kingdom	Recruitment: From 2013–2014. Monitoring period: 6 months	PEN	Form: oral. Three groups: (1) standard care; (2) food-based intervention (increase in calories and protein) and (3) EN supplements 600 kcal and 24 g protein/day.	Assessment at initiation, 3 and 6 months. (1) Record (dietitian) of daily food intake or supplements. Adherence calculation: mean ingested intake, at 3 and 6 months. (2) Anthropometric parameters; (3) Satisfaction and quality of life (no results are shown)	Adherence to interventions = 74% (3 months) and 67% (6 months). 86% of the patients met less than 50% of the requirements of the interventions. Better compliance rates were obtained in food-based intervention versus supplements in all measurements.
Hamza et al. 2015 [20]	Randomized clinical trial	*N*: 37 M/F: 20/17 Age: IG 63 (58–69) CG 67 (63–70)	Pancreatic neoplasia	PH	United Kingdom	Recruitment: 28 months; Monitoring period: 21 days	Preoperative: PEN; Postoperative: EEN	Preoperative period. Form: oral EN. Frequency: 3 EN formula cartons + Normal intake. Postoperative period (7 days). Form: NJ catheter. Frequency: 100 kcal/100 mL up to a target of 25 kcal/kg.	Measurements: 14 days before surgery; on the day of surgery and on days 3 and 7 postsurgery. (1) Journal of compliance: record of quantities ingested 14 days before surgery. (2) Assessment of compliance: daily comparison with the count of the number of containers not consumed	No significant differences in the calories consumed between the two groups. No data on the assessment of compliance.
Bowrey et al. 2015 [21]	Randomized clinical trial	*N* = 41 M/F = 36/5 Age: IG (mean = 64.6 (SD = 8)); CG (mean = 63.1 (SD = 8.7)	Neoplasms of the esophagus or stomach	PNH	United Kingdom	Recruitment: October 2007–June 2009. Monitoring period: 6 months	PEN	Form: JS; Frequency: IG: nocturnal EN administration of 50% energy + protein requirements; CG (standard care): EN only if needed.	Assessment at discharge, at 6 weeks post discharge and at 3 and 6 months postsurgery. (1) Written information on dietary advice and nutritional supplements; (2) Nutritional intake: total energy, proteins, oral nutritional supplements and food by JS; (3) Quality of life. General and specific questionnaires	The results of quality of life were similar between the two groups. Monitoring of adherence to EN was not performed.
Sukkar et al. 2013 [22]	Comparative study	*N*: 22 M/F: 8/14 Age mean: 48.64 (SD = 10.10)	Obese patients	PNH	Italy	Recruitment period: March 2011–October 2011; Monitoring period: 30 days	EEN (10 days); Hypocaloric diet (20 days)	EEN phase: Form: NG: frequency: 10 days EN formula, individual calculation of requirements. Hypocaloric phase: form = oral; frequency: 20 days, caloric deficit of 10% on patient needs	(1) Lifestyle changes, diet and treatments explained by the dietitian at the beginning (2) Technical explanation at the beginning regarding the handling of EN pump; (3) EEN phase: recording by the patient of complications with the feeding tube. Monitoring: at the beginning and at the end of the study.	Three patients (9%) abandoned the study due to low compliance. Monitoring of diet compliance was not performed, only complications of NG tube management
Shirakawa et al. 2012 [23]	Comparative study	*N* = 25 M/F: 19/6 Age, median = 64 years	Pancreatic neoplasia	PH	Japan	Recruitment period: February 2005–November 2006. Monitoring period 5 days	PEN	IG. Form: oral. EN + usual diet 5 days before surgery CG = patients operated without preoperative intake of EN	Assessment of compliance with EN formula: monitoring by the physician during the study period	82.6% (19 patients) fully complied with the protocol of ingestion of EN. Four patients were not compliant.
Sadasivan et al. 2012 [24]	Randomized clinical trial	*N*: 100 M/F: 67/33 Age: Not available	Neoplasia of the head and neck	PNH	India	Recruitment period: 2009–2011. Monitoring period: 6 months	EEN	Form: GS (G1) and NG tube (G2).	Assessment at weeks 1 and 6 and 6 months. (1) Assessment of nutritional status, complications of nutrition and patient satisfaction (pain, feeding tube management, comfort) using the questionnaire [EORTC QLQH&N35].	No documentation or monitoring of intake in terms of calories. Higher satisfaction and lower rate of GS versus NG tube complications at 6 weeks. No comparative data at 6 months.
Kraft et al. 2012 [25]	Prospective, randomized controlled study	*N* = 26 M/F: 10/16 Age: median = 79.8, SD = 7.3	Geriatric patients at risk of malnutrition	PNH	Ireland	Recruitment period: 1 March–31 August 2010. Monitoring period: Not available	PEN	Form: oral route. Frequency: nutritional supplements of 600 kcal.	CG: monitoring at 6 months, measure of nutritional status; IG: telemedicine monitoring. Daily assessment of body weight, number of supplements ingested and health status using telematic questionnaire.	There were no data on the rate of adherence in the IG, nor comparative data between the two groups. Monitoring nutrition and contact with HCP can improve adherence to EN.
Miyata et al. 2012 [26]	Randomized clinical trial	*N* = 91 G1 = 47 M/F = 34/13 Age: mean 62.4 G2 = 44 M/F = 35/9 Age: mean 63.2	Esophagus neoplasia	PNH	Japan	Recruitment period: Not available Monitoring period: 2 17-day cycles of chemotherapy spaced 4 weeks apart	PEN: G1 NPP: G2	G1: omega 3 EN, oral or NG tube. Frequency: G1 and G2 = 600 kcal/day 3 days before and 7 days after 2 17-day cycles of chemotherapy+ oral diet.	Daily consumption of calories (not indicated how it was measured)	Similar calorie intake between the two groups. Similar nutritional indicators between both groups at the end of the study. Six patients from G1 abandoned EN due to its adverse effects
Pohl et al. 2009 [27]	Randomized clinical trial	*N*: 105 M/F: 50/47 Age: 44–91	Type 2 diabetes	PNH	Germany	Recruitment period: June 2004–June 2005. Monitoring period: 84 days	EEN	Form: NG tube or GS Frequency: continuous administration 30 mL (27 kcal)/kg/day to a maximum of 2025 kcal/day. IG: disease-specific EN formula; CG: standard isoenergetic formula	No monitoring of adherence. The presence of adverse effects (tolerability) on days 1, 28, 56 and 84 were measured.	No differences in EN tolerability.
Takagi et al. 2006 [28]	Randomized clinical trial	*N*: 51 M/F: 37/14 Age (mean, SD: IG = 30.8 (11.1) and CG = 28.9 (8.1)	Crohn’s disease	PNH	Japan	Recruitment period; 2002–2005. Monitoring period = 11, 9 months (mean monitoring: 1–28 months, SD = 1.7).	PEN	IG: (1/2 EN + 1/2 normal diet without restriction). Form = EN: oral/intubation according to patient preference Formula = IG: 900–1200 kcal/day elemental diet. CG = normal diet without restriction	Patients were advised on feeding and calculating the daily food intake. (1) At least one visit with clinicians every 3 months: record of adverse effects and anthropometric parameters. Ingestion of EN was not assessed.	Similar adherence in both groups, no concrete data nor specification regarding how it was measured
McGough et al. 2006 [29]	Randomized clinical trial	*N*: 50 M/F: 6/44 Age: (mean, SD) (58, 55–61)	Neoplasia of the pelvis	PNH	United Kingdom	Recruitment period: May 2003–May 2004. Monitoring period: 5 weeks	PEN	Form: Oral route, EN + normal diet. Frequency: different types of formulas and differences in% of daily caloric intake substituted by EN; G1 = 20%; G2 and G4 = 50%; G3 = 75% and G5 = 50%.	(1) Daily filled out by the patient with daily intake, flavor chosen and relevant comments. Collection at the end of treatment. (2) Weekly meeting with dietitian for support and advice on diet. Adherence was determined by evaluating the container counts and weekly diaries of patients	Only three patients complied with the entire treatment (3/50). The number of patients who consumed EN decreased over time: 92% week 1 to 46% week 5. The type of formulation did not affect compliance. In lower prescribed volumes, lower intake was observed.
Piquet et al. 2002 [30]	Comparative study	*N*: 90 M/F = 43/2 (IG), 42/3 (CG). Age = IG: 61 years (SD = 1.5); CG: 59 years (SD = 1.5)	Oropharyngeal neoplasia	PNH	Japan	Recruitment period: September 1998 to September 1999. Monitoring period: 6–7 months	IG: PEN; CG: EN (historical cohort of patients, does not specify EN/PEN)	IG. Form: GS, NG tube or oral route. Frequency: 30 kcal/kg/day, polymeric formula	IG: Initial nutritional assessment before the initiation of radiation therapy: recording of dietary intake, current weight, usual weight, height and Body Mass Index (BMI). Monitoring by dietitian at least three times during radiotherapy treatment. CG: Not specified.	IG: Compliance in 80% of patients, even in alcoholic patients because nutritional support was proposed by medical staff at an early stage as an important component of treatment.
Tsujikawa et al. 2000 [31]	Comparative study	*N*: 20 M/F = 13/7 Age: 28.2 (17–49)	Crohn’s disease	PNH	Japan	Recruitment period: January 1994–1997	Diet rich in omega-3 fatty acids and EN at home; nutritional education	Form: oral route. G1 (omega 3-rich diet) + nutrition education; G2: elemental diet	(1) Dietitian nutrition education prior to discharge and monitoring compliance every 2–4 weeks in the first month. (2) Satisfaction questionnaire for patients with EN delivered when visiting the hospital (periodicity is not specified).	80% of patients did not want to continue taking the elemental diet because it decreased their quality of life. Omega-3 diet + nutritional education improved patient satisfaction, and therefore compliance.
Lawson et al. 2000 [32]	Randomized clinical trial	*N*: 187 (84 IG, 97 CG) M/F: 27/57 Age: (mean, SD) 72 years (40–88)	Patients undergoing orthopedic intervention	PH	United Kingdom	Recruitment period: 18 months. Average monitoring period: 14.4 days	PEN	IG: Form, oral route. Frequency: two oral supplements/day during hospital stay + usual meals. CG: standard diet	(1) Signature by the patient when the supplement was given. Patients, nurses and home health staff recorded the amount ingested for each supplement. (2) Review by dietitian weekly: discussion with the patient regarding progress and recording of total ingested and/or rejected supplements. (3) Record by the patient of food and beverages consumed apart from hospital catering. Adherence: difference between the amount consumed and the amount prescribed.	No patient was 100% adherent. Mean compliance = 14.9%; mean = 0.9%. An average of 6.1 supplements were taken daily, for an average of 6.7 days. Patients took half of the total prescribed volume for half the indicated time. Readjustments of EN: 76% of patients changed the type of supplement, and eight patients decided not to take any. Lower level of compliance in patients with BMI less than 25 kg/m^2^.
Park et al. 1992 [33]	Randomized clinical trial	*N*: 40 M/F: 22/18 Age: 56 years (SD = 4.8) G1 and 65 years (SD = 2.6) G2	Neurological patient with persistent dysphagia	PH	United Kingdom	Monitoring period: 28 days	EEN	Form: G1: NG tube; G2: GS. Frequency: infusion 24 h. Volumes adjusted to the needs of each patient.	(1) Measured daily record of the remnant in the feeding tube. (2) Adherence = amount received, % above the prescribed. (3) EN Acceptance Measurement Questionnaire completed by patient or family. (4) Treatment failure	93% (SD = 2%) adherence G2 versus G1 (55% (SD = 4%)). Increased weight gain in the first week of treatment, G2 versus G1. 95% G1 patients had treatment administration failures, and none in G2.

Anti-TNF: anti-Tumor Necrosis Factor; BMI = Body mass index; CG = Control group; CI = Confidence interval; G = group; h = hour; EEN = Exclusive enteral nutrition; EN = enteral nutrition; GS = Gastrostomy; HCP = Health Care Professionals; H/M = male/female; IG = intervention group; JS = Jejunostomy; MEP: Manutrition Energy Protein; NC = Not recorded; ND = Nasoduodenal; NG = Nasogastric; PEN = Partial enteral nutrition; PH = Hospitalized patient; PNH = Non-hospitalized patient; SD: Standard Desviation; PH = Hospitalized patient; PNH = Non-hospitalized patient; SD: Standard Desviat.

**Table 2:** Assessment of study quality according to the 25-item CONSORT guidelines.

	1	2	3	4	5	6	7	8	9	10	11	12	13	14	15	16	17	18	19	20	21	22	23	24	25	Total
Hirai et al. 2019 [11]	0.5	1	0.5	1	0	0.5	0.5	0	0	0	0	0.5	0.5	0.5	1	1	0.5	0	1	1	0	1	0	0	0	7 28%
Wall et al. 2018 [12]	0.5	1	0	1	1	0.5	0	0	0	0	0	0.5	1	0	1	1	0.5	0	1	1	1	1	0	0	1	11 44%
Benton et al. 2018 [13]	0.5	1	1	1	1	0.5	0.5	0.5	0	0	0	0.5	1	0	1	1	0.5	1	0	1	0	1	0	0	1	11 44%
Deane et al. 2018 [14]	1	1	0.5	0.5	1	0.5	0	1	0	0	0.5	0.5	1	0.5	1	1	0.5	0	1	1	0	1	0	1	0	11 44%
Brown et al. 2017 [15]	0.5	1	0	0	1	0	0	0	0	0	0	0.5	1	0.5	1	1	0.5	0	1	1	0	1	0	0	1	9 36%
Healy et al. 2017 [16]	0	0.5	0	1	1	0.5	0.5	1	0	0	0.5	0.5	1	0.5	1	1	0.5	0	1	0	0	1	0	0	0	11.5 46%
Harvey et al. 2016 [17]	1	1	0.5	1	1	0.5	1	1	1	1	0.5	1	1	0.5	1	1	0.5	1	1	1	1	1	1	1	1	20 80%
Zhao 2015 [18]	0.5	0.5	0	0.5	1	0.5	0	0	0	0	0	0.5	0	0	0	0	0.5	0	1	0	1	1	0	0	0	7 28%
Stow et al. 2015 [19]	1	1	0.5	1	1	0.5	0.5	1	1	1	0.5	0.5	1	1	1	1	0.5	0	1	1	1	1	1	1	1	21 84%
Hamza et al. 2015 [20]	1	1	0.5	0.5	1	0.5	0.5	1	0	0	0	1	1	0	1	1	0.5	0	0	1	0	1	0	0	0	12.5 50%
Bowrey et al. 2015 [21]	1	1	0.5	0.5	0	1	0.5	1	0	0.5	0	0.5	1	0.5	1	1	0	0	1	0	1	1	0	0	1	14 56%
Sukkar et al. 2013 [22]	0	1	0	0.5	1	0.5	0	0	0	0	0.5	0.5	1	0.5	1	0	0.5	0	1	0	1	1	0	1	1	12 48%
Shirakawa et al. 2012 [23]	0.5	1	0	0.5	1	0.5	0	0	0	0	0	0.5	0.5	0.5	1	1	0.5	0	1	0	1	1	0	0	0	11.5 46%
Sadasivan et al. 2012 [24]	0	1	1	0.5	1	0.5	0.5	1	0	0	0	0.5	1	0.5	1	1	0.5	0	1	1	0	1	0	0	0	13 52%
Kraft et al. 2012 [25]	0	1	0.5	0.5	1	0.5	0.5	0.5	0	0	0	0	1	0.5	1	1	0	0	0	0	1	1	0	0	1	11 44%
Miyata et al. 2012 [26]	1	1	0	0.5	1	0.5	0	0.5	0	1	0	0.5	1	0.5	1	1	0.5	0	1	1	0	1	0	0	1	14 56%
Pohl et al. 2009 [27]	1	0.5	0.5	1	1	0.5	0.5	0.5	1	1	1	0.5	1	1	1	1	1	0	1	1	1	1	0	0	0	18 72%
Takagi et al. 2006 [28]	1	1	0	1	1	0.5	0.5	1	1	1	0.5	0.5	1	1	1	1	0.5	0	1	1	1	1	0	0	0	17.5 70%
McGough et al. 2006 [29]	0.5	1	0.5	1	1	0.5	0.5	1	1	1	0	1	0.5	0	1	1	0	1	1	0	1	1	0	0	1	16.5 66%
Piquet et al. 2002 [30]	0	1	0	0	1	0.5	0	0	0	0	0	0.5	0.5	0.5	1	1	0.5	0	1	0	1	1	0	0	0	9.5 38%
Tsujikawa et al. 2000 [31]	0	1	0	1	1	0.5	0	0	0	0	0	0.5	0	0	0	0	0.5	1	0	1	0	1	0	0	0	7.5 30%
Lawson et al. 2000 [32]	0.5	1	0	1	1	0	0	0	0	0	0	0.5	0.5	0	0	0	0.5	0	1	0	0	1	0	0	0	7 28%
Park et al. 1992 [33]	1	0.5	0.5	1	1	0.5	0.5	0.5	1	0	0	1	1	1	1	1	0.5	1	1	0	1	1	0	0	1	17 68%
